# Analysis of Risk Factors for Death in the Coronavirus Disease 2019 (COVID-19) Population: Data Analysis from a Large General Hospital in Anhui, China

**DOI:** 10.7759/cureus.60069

**Published:** 2024-05-10

**Authors:** Shi Wei, Wu Xiaqin, Liu Liwei, Zhang Fasu, Pan Ying, Tian Pingping, Yu Furong

**Affiliations:** 1 Medical Laboratory Science, Anhui Medical College, Hefei, CHN; 2 Medical Laboratory, Anqing Center, Anhui Medical University, Anqing, CHN; 3 Immunology, Anhui Medical College, Hefei, CHN

**Keywords:** hematological examination, clinical chemistry, severity, risk factors, covid-19

## Abstract

During the coronavirus disease 2019 (COVID-19) pandemic, clinical prevention, early diagnosis, and hematological monitoring were challenging areas. This study aims to compare risk factors and hematological and biochemical data in non-survivor group patients with COVID-19 versus survivor group patients. A total of 204 patients with COVID-19 were selected as research subjects from December 2022 to January 2023. We analyzed the age, sex, time from onset to admission, and laboratory test indicators upon admission. The differences between surviving and deceased patients and mortality-related risk factors were examined. Among the 204 patients, 168 survived, whereas 36 died during hospitalization. Significant differences were observed between the two groups with COVID-19 across various factors, including age (p < 0.0001), WBC count (p < 0.0001), RBC count (p < 0.05), neutrophils (p < 0.0001), lymphocytes (p < 0.05), mean corpuscular hemoglobin concentration (MCHC) (p < 0.0001), RBC distribution width-standard deviation (RDW-SD) (p < 0.0001), RBC distribution width coefficient of variation (RDW-CV) (p < 0.0001), aspartate aminotransferase (AST) (p < 0.05), albumin (ALB) (p < 0.0001), creatinine (CR) (p < 0.0001), uric acid (UA) (p < 0.0001), blood urea nitrogen (BUN) (p < 0.0001), plasma thrombin time (TT) (p < 0.05), prothrombin time (PT) (p < 0.0001), and D-dimer (p < 0.0001). Multivariate logistic analysis revealed that older age, CR, UA, and ALB were independent factors associated with death (p < 0.05). Elderly patients with underlying diseases, abnormal routine blood test indices, and abnormal renal function and coagulation indices are at an increased worse prognosis and should be identified early. Age, UA, CR, and ALB can be used as predictors to assess the worse prognosis in the hospital.

## Introduction

The novel coronavirus disease 2019 (COVID-19) [1] is an acute respiratory tract infection that was first identified in Wuhan and has caused a global pandemic since 2019. As of February 1, 2023, there have been more than 753 million confirmed cases and over 6.8 million deaths reported worldwide [2]. The main circulating strain in China was Omicron, along with mutations of the coronavirus. COVID-19 can present with a range of clinical manifestations, from asymptomatic infection to severe acute respiratory syndrome, septic shock, and acute respiratory distress syndrome (ARDS) [3]. Although the mortality rate is not as high, the increasing number of COVID-19 cases in China, with its large population base and significant aging population, poses challenges in terms of healthcare resources. Critically ill patients, especially those who are elderly or have underlying diseases, are at higher risk of death.

Recent studies in specialty medical journals have found that elderly patients or those with obesity, cardiovascular disease, diabetes, cancer, hematological malignancy, chronic kidney disease, and chronic obstructive pulmonary disease (COPD) have a higher risk of death compared to the general population [4,5].

Currently, real-time polymerase chain reaction (RT-PCR) is the best noninvasive and rapid diagnostic method for detecting the coronavirus [6,7]. However, RT-PCR has limitations in sensitivity and cannot assess the activity and severity of COVID-19 in patients [8]. Therefore, identifying blood biomarkers associated with the risk of death can be helpful in predicting clinical outcomes and monitoring treatment efficacy. Several biomarkers, including WBC count, lymphocyte count, D-dimer, RBC distribution width (RDW), and ALB have been shown in various studies to predict mortality [9-12].

The aim of this study is to analyze the clinical characteristics and prognostic risk factors of hospitalized COVID-19 patients in the early stages of the epidemic. Hematologic and biochemical abnormalities will be retrospectively analyzed in COVID-19 patients with severe and non-severe cases, providing valuable insights for the diagnosis and treatment of severe COVID-19 patients.

## Materials and methods

Patients and study design

This study retrospectively analyzed the clinical records of 204 COVID-19 patients treated at prestigious hospitals in Anhui, China, from December 2022 to January 2023. The diagnosis of COVID-19 was primarily based on etiology, with RT-PCR (by Shanghai Bojie Medical Technology Co. Ltd., Pudong, China) and lung computed tomography (CT) being essential diagnostic methods. The inclusion criteria for this study were patients above 18 years old and not pregnant, according to the clinical diagnostic criteria for COVID-19. Exclusion criteria were pernicious tumor patients, radiation and chemotherapy treatment of patients, pregnancy, general systemic disease, severe infection, hematological disease, and incomplete clinical and imaging data. The patients were divided into two groups: survivors (168 patients) and non-survivors (36 patients).

Data collection

Patient demographics including age, sex, and time from onset to admission were collected, and laboratory examinations were conducted upon admission. Retrospective analysis of laboratory indexes was performed, including results from routine blood analysis (WBC count, RBC count, neutrophils, lymphocytes, hemoglobin, platelets; hematocrit (HCT); mean corpuscular volume (MCV); mean corpuscular hemoglobin (MCH); mean corpuscular hemoglobin concentration (MCHC); RBC distribution width-standard deviation (RDW-SD); RBC distribution width coefficient of variation (RDW-CV)), liver function (aspartate transaminase (AST), alanine transaminase (ALT), total bilirubin (TBIL), total protein (TP), ALB), renal function (creatinine (CR), uric acid (UA), blood urea nitrogen (BUN)), coagulation function (plasma prothrombin time (PT), activated partial thromboplastin time (APTT), plasma thrombin time (TT), fibrinogen (FIB)), and D-dimer. A comprehensive analysis was conducted to assess the differences between the survival and non-survival groups and identify worse prognosis factors.

Statistical analysis

Statistical analysis was performed using Statistical Product and Service Solutions (SPSS) (version 26; IBM SPSS Statistics for Windows, Armonk, NY). Data that followed a normal distribution were analyzed using a t-test, while non-normally distributed data were analyzed using medians and quartiles, and the Mann-Whitney U-test was employed. Binary logistic regression analysis was conducted to determine independent risk factors for mortality. A Spearman's rank correlation analysis was used to assess correlations between different independent factors. Furthermore, the diagnostic value of the risk factors in predicting death in severe patients was evaluated using a receiver operating characteristic (ROC) curve analysis. The ROC curve was generated, and the area under the curve (AUC) was calculated. The cutoff value was determined using the Youden index. Statistical significance was considered when the P value was less than 0.05.

## Results

The study included a total of 204 COVID-19 patients, consisting of 66 females and 138 males, from December 2022 to January 2023. The patients were divided into two groups based on their prognosis: the survivor (168 cases) and non-survivor groups (36 cases). The median age of the survivor group ranged from 55 to 77 years old, with a mean age of 68 years old. The non-survivor group had a median age ranging from 71 to 83 years old, with a mean age of 80 years old. There was a significant difference in age distribution in the two groups (p < 0.0001), as shown in Table 1. However, there were no significant differences in sex ratio or hospital days between the two groups (p > 0.05).

**Table 1:** Baseline characteristics of the patients ALB: albumin; ALT: alanine transaminase; APTT: activated partial thromboplastin time; AST: aspartate transaminase; BUN: blood urea nitrogen; CR: creatinine; FIB: fibrinogen; HCT: hematocrit; IU: international units; MCH: mean corpuscular hemoglobin; MCHC: mean corpuscular hemoglobin concentration; MCV: mean corpuscular volume; M-F: male-female; PLT: platelet; PT: prothrombin time; RDW-CV: RBC distribution width coefficient of variation; RDW-SD: RBC distribution width-standard deviation; TBIL: total bilirubin; TP: total protein; TT: thrombin time; UA: uric acid

Variable	Survivor	Non-survivor	P value
Age (y)	68 (55-77)	80 (71-83)	<0.0001
Sex (M-F)	110:58	28:8	0.152
Hospital day	11.00 (8.00-15.00)	13.00 (7.5-17)	0.342
TP (g/L)	66.55±7.18	66.29±9.00	0.217
ALB (g/L)	37.9 (34.55-40.5)	32.90 (30.35-35.25)	<0.0001
ALT (IU/L)	23 (13-35)	21.50 (14-45.5)	0.756
AST (IU/L)	31.00 (23.75-47.25)	40.50 (30.25-56.75)	<0.05
TBIL (μmol/L)	9.95 (5.93-13.4)	10.65 (7.15-15.95)	0.143
BUN (mmol/L)	5.73 (4.40-8.60)	14.15 (9.58-20.68)	<0.0001
CR (μmol/L)	85 (59-119)	148.00 (86.50-298.75)	<0.0001
UA (μmol/L)	278 (205.5-355)	400.00 (292.50-540.50)	<0.0001
PT (s)	12.7 (12-13.7)	13.70 (12.75-15.05)	<0.0001
APTT (s)	38.05 (33.58-41.3)	38.00 (33.20-44.30)	0.461
TT (s)	16.90 (15.80-18.30)	17.70 (16.50-19.68)	<0.05
FIB (g/L)	4.95 (3.83-5.88)	5.41 (4.27-6.46)	0.129
D-dimer (μg/mL)	0.85 (0.44-1.66)	2.07 (1.43-3.49)	<0.0001
RDW-CV (%)	13.2 (12.7-13.7)	14.20 (13.70-15.45)	<0.0001
RDW-SD	45 (42.8-47.7)	48.70 (45.30-52.13)	<0.0001
Hemoglobin (g/L)	123.51±19.97	115.19±23.98	0.119
HCT (%)	37.4 (32.7-40.3)	37.30 (31.03-39.08)	0.351
MCV (fl)	91 (87.9-94.5)	92.20 (87.23-95.98)	0.607
MCH (pg)	30.7 (29.4-31.9)	30.00 (28.43-31.95)	0.153
MCHC (g/L)	336.00 (329.00-324.00)	326.00 (319.50-336.75)	<0.0001
WBC (109/L)	5.64 (4.25-8.07)	9.36 (6.19-13.43)	<0.0001
RBC (1,012/L)	4.05±0.67	4.00±1.01	<0.05
Neutrophil (109/L)	4.00 (2.80-6.80)	8.25 (5.05-11.50)	<0.0001
Lymphocyte (109/L)	0.90 (0.50-1.30)	0.65 (0.40-1.00)	<0.05
PLT (109/L)	159.00 (112.00-211.00)	151.00 (107.25-210.00)	0.732

Furthermore, various hematological, blood biochemical, coagulation function, and D-dimer parameters were evaluated. The AST, UA, CR, UA, PT, TT time, and D-dimer levels were significantly higher in the non-survivor group compared to the survivor group. Conversely, the serum ALB levels were lower in the non-survivor group. These differences were statistically significant (p < 0.05), as shown in Table 1. Additionally, the non-survivor group exhibited significantly higher WBC and neutrophil counts, as well as higher RDW-CV and RDW-SD, compared to the survivor group. Moreover, the non-survivor group had significantly lower blood cell and lymphocyte counts, as well as lower MCHC, compared to the survivor group. However, there were no significant differences in other data between the two groups.

It was determined through single-factor regression analysis that age, WBC count, neutrophil count, ALB, urea nitrogen, CR, UA, RDW-CV, RDW-SD, hemoglobin, MCH, and MCHC were independent risk factors associated with death from COVID-19 (p < 0.05). However, the other variables were not found to be significantly associated with the risk of death (p > 0.05) (Table 2). Furthermore, multiple regression analysis was performed on the factors identified through binary logistic regression. The results showed that age, low ALB levels, and elevated urea nitrogen and CR levels were the most important risk factors for death from COVID-19, as shown in Table 2.

**Table 2:** Univariate and multiple regression logistic regression analysis to identify risk factors associated with mortality in patients with COVID-19 ALB: albumin; ALT: alanine transaminase; APTT: activated partial thromboplastin time; AST: aspartate transaminase; BUN: blood urea nitrogen; CI: confidence interval; CR: creatinine; FIB: fibrinogen; HCT: hematocrit; IU: international units; MCH: mean corpuscular hemoglobin; MCHC: mean corpuscular hemoglobin concentration; MCV: mean corpuscular volume; OR: odds ratio; PLT: platelet; PT: prothrombin time; RDW-CV: RBC distribution width coefficient of variation; RDW-SD: RBC distribution width-standard deviation; TBIL: total bilirubin; TP: total protein; TT: thrombin time; UA: uric acid

Variables	Univariate		Multivariate	
	OR (95% CI)	P value	OR (95% CI)	P value
Age	1.067 (1.031,1.104)	<0.001	0.893 (0.845,0.943)	<0.001
Hospital day	1.040 (0.990,1.093)	0.121		
TP (g/L)	0.995 (0.948,1.044)	0.848		
ALB (g/L)	0.867 (0.808,0.931)	<0.001	1.152 (1.027,1.292)	<0.05
ALT (IU/L)	1.002 (0.998,1.006)	0.365		
AST (IU/L)	1.000 (0.999,1.022)	0.536		
TBIL (μmol/L)	1.010 (0.918,1.040)	0.499		
BUN (mmol/L)	1.019 (1.049,1.135)	<0.001		
CR (μmol/L)	1.002 (1.000,1.003)	<0.05	0.997 (0.994,1.000)	<0.05
UA (μmol/L)	1.004 (1.002,1.004)	<0.001	0.995(0.991,1.000)	<0.05
PT (s)	1.240 (1.042,1.475)	<0.05		
APTT (s)	1.006 (0.982,1.030)	0.639		
TT (s)	0.999 (0.974,1.024)	0.913		
FIB (g/L)	1.191 (0.981,1.444)	0.077		
D-dimer (μg/mL)	1.010 (0.997,1.024)	0.124		
RDW-CV (%)	1.841 (1.379,2.457)	<0.001		
RDW-SD	1.136 (1.048,1.230)	<0.05		
Hemoglobin (g/L)	0.918 (0.964,0.998)	<0.05		
HCT (%)	0.961 (0.908,1.018)	0.180		
MCV (fl)	0.964 (0.917,1.014)	0.154		
MCH (pg)	0.821 (0.714,0.944)	<0.05		
MCHC (g/L)	0.925 (0.891,0.961)	<0.001		
WBC (10^9/L)	1.158 (1.065,1.259)	<0.001		
RBC (10^12/L)	0.922 (0.567,1.499)	0.742		
Neutrophil (10^9/L)	1.203 (1.095,1.321)	<0.001		
Lymphocyte (10^9/L)	1.001 (0.625,1.537)	0.998		
PLT (10^9/L)	1.001 (0.996,1.005)	0.751		

Correlation analysis was conducted using Spearman rank correlation to examine the relationship between the independent factors and other laboratory indexes. The results indicated that age, ALT, TBIL, UA, RDW-CV, RDW-SD, WBC count, and neutrophil count were positively correlated. Conversely, age showed a negative correlation with total protein and lymphocyte count (Table 3-5). In terms of ALB, it was found to be significantly associated with total protein, hemoglobin, and HCT. However, it exhibited a negative correlation with hospital day, AST, TBIL, UA, PT, APTT, FIB, RDW-CV, RDW-SD, MCV, MCH, WBC count, RBC count, and neutrophil count (Tables 3-5). Furthermore, CR showed a positive correlation with hospital day, AST, TBIL, UA, FIB, RDW-CV, RDW-SD, WBC count, and neutrophil count. However, it had a negative correlation with hemoglobin, HCT, MCHC, and RBC count (Table 3-5). The variables hospital day, total protein, AST, UA, PT, FIB, RDW-CV, RDW-SD, WBC count, neutrophil count, and lymphocyte count were positively associated with urea nitrogen. Conversely, MCV, MCH, and MCHC showed a significant negative correlation (Tables 3-5).

**Table 3:** The correlation coefficient between the independent factors and hospital day, liver function, and uric acid A P < 0.05 was considered statistically significant between the two data. AST: aspartate aminotransferase; ALT: alanine transaminase; TBIL: total bilirubin

	Hospital day	Total protein	ALT	AST	TBIL	Uric acid
Age (y)	0.06	-0.15^a^	-0.06	0.15^a^	0.20^a^	0.20^a^
Albumin (g/L)	-0.19^a^	0.43^a^	0.05	-0.16^a^	-0.18^a^	-0.25^a^
Creatinine (μmol/L)	0.18^a^	0.06	0.01	0.20^a^	0.16^a^	0.79^a^
Urea nitrogen (μmol/L)	0.20^a^	0.14^a^	0.06	0.18^a^	0.13	0.62^a^

**Table 4:** The correlation coefficient between the independent factors with blood coagulation examination a: P < 0.05 was considered statistically significant between the two data PT: prothrombin time; APTT: activated partial thromboplastin time; TT: thrombin time; FIB: fibrinogen; D-D: D-dimer

	PT	APTT	TT	FIB	D-D
Age (y)	0.05	0.10	0.06	0.09	0.07
Albumin (g/L)	-0.22^a^	-0.17^a^	-0.14	-0.30^a^	-0.09
Creatinine (μmol/L)	0.04	0.14	0.11	0.21^a^	0.06
Urea (μmol/L) nitrogen	0.26^a^	0.10	0.02	0.23^a^	0.05

**Table 5:** The correlation coefficient between the independent factors with routine blood test A P < 0.05 was considered statistically significant between the two data. RDW-CV: RBC distribution width-coefficient of variation; RDW-SD: RBC distribution width-standard deviation; HCT: hematocrit; MCV: mean corpuscular volume; MCH: mean corpuscular hemoglobin; MCHC: mean corpuscular hemoglobin concentration; PLT: platelet

	RDW-CV	RDW-SD	Hemoglobin	HCT%	MCV	MCH	MCHC	PLT	WBC	RBC	Neutrophil	Lymphocyte
Age (y)	0.18^a^	0.29^a^	-0.10	-0.10	0.10	0.08	-0.03	-0.09	0.15^a^	-0.11	0.18^a^	-0.19^a^
Albumin (g/L)	-0.35^a^	-0.37^a^	0.15^a^	0.14^a^	-0.15^a^	-0.13	0.01	-0.04	-0.28^a^	0.20^a^	-0.31^a^	0.02
Creatinine (μmol/L)	0.18^a^	0.22^a^	-0.42^a^	-0.39^a^	0.04	-0.03	-0.14^a^	-0.01	0.20^a^	-0.37^a^	0.24^a^	-0.09
Urea nitrogen(μmol/L)	0.35^a^	0.14^a^	-0.03	0.01	-0.18^a^	-0.24^a^	-0.18^a^	-0.08	0.34^a^	0.09	0.32^a^	0.20^a^

**Figure 1:**
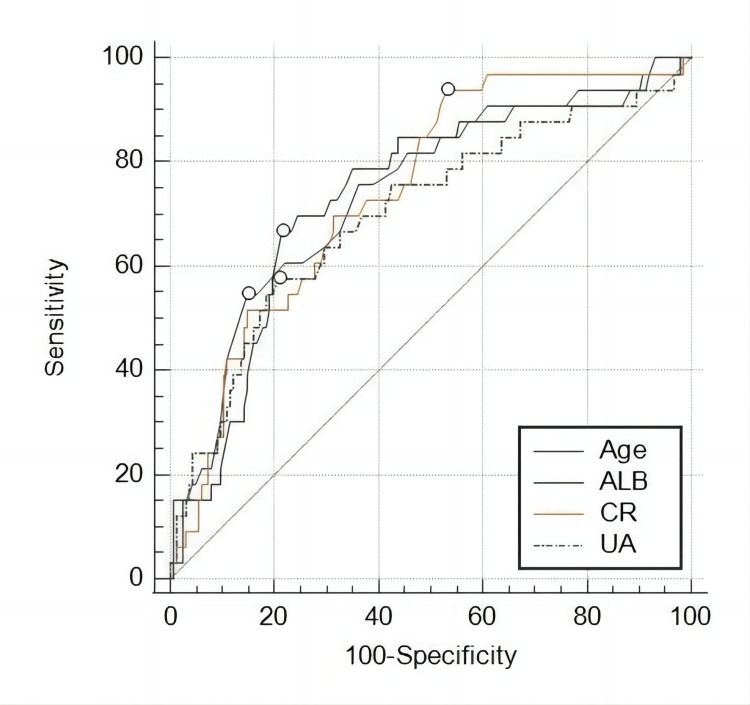
ROC curve comparing the diagnostic validity of age, ALB, CR, and UA in the prediction of mortality among COVID-19 patients ALB: albumin; CR: creatinine; UA: uric acid

The area under the ROC curves was calculated to evaluate the predictive ability of age, ALB, CR, and BUN for mortality from COVID-19. The AUC value for age was 0.743 (95% CI= 0.675 to 0.802, P < 0.05), with a sensitivity of 54.55% and specificity of 85.19%. The cutoff value for age was determined to be 79 years old. For ALB, the AUC value was 0.734 (95% CI= 0.666 to 0.795, P < 0.05), with a cutoff value of 33.7 g/L. The sensitivity and specificity were 66.67% and 78.4%, respectively. In the case of CR, the AUC value was 0.744 (95% CI, 0.676-0.803; p < 0.05), with a sensitivity of 93.94% and specificity of 46.91%. The cutoff value for CR was 80.00 μmol/L. Moreover, the mountain curve analysis indicated that the model had a sensitivity of 57.58%, a specificity of 79.01%, and an AUC value of 0.701 (95% CI= 0.632 to 0.765, p < 0.05). The cutoff value for BUN was determined to be 373.00 μmol/L. The ROC curve and corresponding AUC values are presented in Figure 1 and Table 6. 

**Table 6:** The ROC diagnostic model of the risk factors for death AUC: area under the curve; ALB: albumin; SE: serum electrolytes

Variables	AUC (95% CI)	Sensitivity (%)	Specificity (%)	Youden index J	Cutoff value	SE
Age (y)	0.743(0.675 to 0.802)	54.55	85.19	0.40	>79.00	0.049
ALB (g/L)	0.734(0.666 to 0.795)	66.67	78.40	0.45	≤33.70	0.049
Creatinine (μmol/L)	0.744(0.676 to 0.803)	93.94	46.91	0.41	>80.00	0.044
Urea nitrogen (μmol/L)	0.701(0.632 to 0.765)	57.58	79.01	0.37	>373.00	0.055

## Discussion

Since December 2019, COVID-19 has spread worldwide, causing diverse global epidemic situations and various responses from different countries. Although the overall mortality rate of COVID-19 is not high, certain populations, particularly those with severe pneumonia, ARDS, acute kidney injury, and acute heart failure, have a higher risk of death [13]. Therefore, early detection and diagnosis of high-risk patients are crucial in reducing the mortality rate among severe cases. Analyzing changes in hematologic examinations in COVID-19 patients can help in assessing disease progression and prognosis.

In our study, we collected clinical data from 204 COVID-19 patients between December 2022 and January 2023 and divided them into survivor and non-survivor groups. The results of blood routine analysis showed that the non-survivor group had significantly higher counts of WBC, neutrophils, RDW-CV, and RDW-SD, and significantly lower levels of MCHC, RBC, and lymphocytes. These findings are consistent with previous research indicating that severe COVID-19 patients may experience more intense inflammatory reactions, leading to increased WBC and neutrophil counts [14-16]. The elevated neutrophil count may contribute to the development of cytokine storms, which can be fatal for critically ill patients [17]. Additionally, increased RDW levels have been identified as independent predictors of COVID-19 progression [18,19]. There are various reasons for increased RDW levels. First, an elevation in systemic inflammation might lead to increased RDW levels in COVID-19 patients. Furthermore, some COVID-19 patients experience disturbances in their internal environment, which might contribute to a shortened RBC survival, resulting in increased RDW. The decreased RBC count and MCHC suggest a potential worsening of anemia in COVID-19 patients. Furthermore, both survivor and non-survivor groups had lower lymphocyte counts compared to healthy individuals, indicating a close association between lymphocyte count decline and COVID-19 severity [20-22].

Coagulation disorders were more pronounced and prolonged in the non-survivor group, as evidenced by significantly elevated PT, TT, and D-dimer levels. Similar studies have indicated that PT and D-dimer levels reflect a hypercoagulable state in COVID-19 patients [23,24]. COVID-19, as an infectious disease, leads to an inflammatory response syndrome. The immune damage it causes is systemic. These coagulation disorders are associated with complex pathophysiology, which is not yet fully understood. The pathogenesis may be related to the development of circulating anticoagulants or impaired synthesis of clotting factors. Elevated D-dimer levels are associated with sepsis-induced coagulopathy and an increased risk of thromboembolism in severe COVID-19 cases [25,26]. Therefore, timely and dynamic monitoring of PT, TT, and D-dimer levels is crucial for preventing thromboembolic events [24].

Biochemical abnormalities in liver function, such as low levels of ALB and increased AST, were found to be associated with mortality. However, the changes in ALT, TP, and TBIL levels did not show significant differences between the two groups. These results are consistent with previous research indicating that elevated AST levels serve as a risk factor for COVID-19 mortality because of immune-mediated inflammatory responses and cytotoxic T cell activity [27,28]. Serum ALB, an acute phase reactant and a marker of inflammation, was significantly correlated with total protein, hemoglobin, and HCT levels, but it showed negative correlations with hospital day, AST, TBIL, UA, PT, APTT, FIB, RDW-CV, RDW-SD, MCV, MCH, WBC count, RBC count, and neutrophil count [29-31]. Serum ALB levels below 33.7 g/L were found to be a significant risk factor for mortality, as indicated by the ROC curve analysis with an AUC value of 0.73, sensitivity of 66.67%, and specificity of 78.4%.

COVID-19 can affect multiple vital organs in addition to the lungs, such as the liver, heart, and kidneys [32]. Studies have shown a significantly higher risk of death in COVID-19 patients with chronic kidney disease or acute kidney injury [4,33-35]. In line with these studies, our findings demonstrated significantly elevated levels of CR, BUN, and UA in the non-survivor group, indicating the impact of renal dysfunction on poor outcomes in COVID-19 patients. In the multivariate logistic regression analysis, older age was identified as an important risk factor for severe COVID-19 cases. Numerous studies have consistently shown that advanced age is associated with poor outcomes in COVID-19 patients [14,36-38]. The mortality risk in severe COVID-19 patients is increased when age exceeds 79 years, ALB is below 33.7 g/L, CR exceeds 80 μmol/L, and UA exceeds 373 μmol/L.

This study has some limitations, including a relatively small sample size and potential bias in the analysis because of the inclusion of different studies. Additionally, healthcare professionals may have limitations in understanding the characteristics of severe COVID-19 patients, leading to variations in treatment options. Despite these limitations, this study provides valuable insights into the mortality profile of COVID-19 patients and contributes to evidence-based decision-making in allocating limited medical resources.

## Conclusions

While clinical hematological parameters may not assess the severity, our findings show that some parameters are independent predictors for evaluating patient outcomes. For example, older age, low levels of ALB, elevated CR, and increased UA are significant. Therefore, monitoring liver and kidney function, along with routine blood tests and coagulation assessments, can provide valuable information for early diagnosis and prognosis prediction. Further research is needed to validate these findings and to gain a better understanding of the factors influencing mortality in COVID-19 patients.
